# High-Dose Prophylactic Anticoagulation for COVID-19 Pneumonia: A Review of Benefits and Risks

**DOI:** 10.7759/cureus.37705

**Published:** 2023-04-17

**Authors:** Yeshu Kattakola, Roshan Prasad, Ranjana Sharma, Mayur B Wanjari

**Affiliations:** 1 Internal Medicine, Jawaharlal Nehru Medical College, Datta Meghe Institute of Higher Education and Research, Wardha, IND; 2 Medical Surgical Nursing, Srimati Radhikabai Meghe Memorial College of Nursing, Datta Meghe Institute of Higher Education and Research, Wardha, IND; 3 Research and Development, Jawaharlal Nehru Medical College, Datta Meghe Institute of Higher Education and Research, Wardha, IND

**Keywords:** risks and benefits, patient selection, thrombotic events, pneumonia, prophylactic anticoagulation, covid-19

## Abstract

The COVID-19 pandemic has had a devastating impact on a global scale, causing significant morbidity and mortality. The virus affects multiple organ systems, including the respiratory, cardiovascular, and coagulation systems, leading to severe pneumonia in some patients. Moreover, COVID-19 patients with severe pneumonia have a high incidence of thrombotic events, which can result in significant morbidity and mortality. Given the potential benefits of anticoagulation therapy in COVID-19 patients with thrombotic complications, recent studies have proposed high-dose prophylactic anticoagulation (HD-PA) therapy as a potential treatment option. In fact, some studies have suggested that HD-PA therapy may be more effective in reducing thrombotic events and mortality rates than other treatment options. This review aims to provide a comprehensive overview of the benefits and risks of HD-PA therapy for COVID-19 pneumonia patients. By synthesizing and analyzing the latest available research, we highlight patient selection criteria and discuss the optimal dosage, duration, and timing of therapy. Additionally, we review the potential risks associated with HD-PA therapy and provide recommendations for clinical practice. Ultimately, this review provides valuable insights into the use of HD-PA therapy in COVID-19 pneumonia patients and paves the way for further research in this critical area. By exploring the benefits and risks of this treatment option, we hope to provide healthcare professionals with the information they need to make informed decisions about the best course of treatment for their patients.

## Introduction and background

COVID-19, caused by the severe acute respiratory syndrome coronavirus 2 (SARS-CoV-2), has had a devastating impact worldwide, leading to high levels of morbidity and mortality. While the disease primarily affects the respiratory system, much research has revealed that COVID-19 can also cause damage to other vital organs, including the cardiovascular and coagulation systems. Among COVID-19 patients, those with severe pneumonia have a higher risk of developing thrombotic complications (TCs), such as deep vein thrombosis and pulmonary embolism, which can result in severe morbidity and mortality [1]. Suh et al. conducted a meta-analysis with 27 studies that included 3342 patients with COVID-19. The pooled incidence rates of PE and DVT were 16.5% and 14.8%, respectively. PE was more frequently found in patients who were admitted to the ICU (24.7%) vs. those not admitted to the ICU (10.5%). DVT was present in 42% of patients with PE [2].

Given the high incidence of thrombotic events among COVID-19 pneumonia patients, anticoagulation therapy has emerged as a potential treatment option. While standard prophylactic anticoagulation therapy has been shown to lower the risk of venous thromboembolism in COVID-19 patients, high-dose prophylactic anticoagulation (HD-PA) therapy may be a more effective approach for these patients, with a greater reduction in thrombotic events and mortality rates [3]. Labbé et al. conducted a randomized control trial with 334 individuals and found that the use of HD-PA and standard-dose prophylactic anticoagulation (SD-PA) had similar probabilities of favorable outcomes (47.3% vs. 52.7%). However, they found that HD-PA use significantly reduced thrombosis compared with SD-PA (absolute difference, -14.7) and that HD-PA resulted in significantly better net clinical outcomes by decreasing the risk of de novo thrombosis [4].

The purpose of this review article is to provide a comprehensive overview of the benefits and risks associated with HD-PA therapy for COVID-19 pneumonia patients. By studying and considering the up-to-date existing studies, we aim to assist healthcare professionals in making informed decisions about the use of this therapy for their patients.

## Review

Material and method

In our literature search on HD-PA therapy for COVID-19 pneumonia, we used keywords like "risks and benefits," "patient selection," "thrombotic events," "pneumonia," "prophylactic anticoagulation," and "COVID-19." We searched various electronic databases and manually screened reference lists of relevant articles. We selected high-quality studies focusing on HD-PA therapy in COVID-19 pneumonia patients while excluding studies on other types of anticoagulation therapy or non-COVID-19 patients. Two independent authors assessed the studies for relevance, and disagreements were resolved through discussion and consensus.

A brief history of anticoagulation therapy in COVID-19

Anticoagulation therapy has become a crucial treatment approach for COVID-19 since the early stages of the pandemic, primarily due to the high incidence of TCs observed in infected patients. As understanding of the disease and its complications has evolved, anticoagulation therapy has expanded beyond critically ill patients, which was the initial focus of studies [5]. Further research has demonstrated that prophylactic anticoagulation therapy can also significantly reduce the incidence of thrombotic events in hospitalized COVID-19 patients. As a result, prophylactic anticoagulation therapy has become an integral part of the standard of care for COVID-19 patients, particularly those at high risk of thrombosis [6].

The use of prophylactic anticoagulation therapy in COVID-19 patients has shown promising results in reducing the incidence of TCs, which are a significant cause of morbidity and mortality in COVID-19 patients. The pro-thrombotic state observed in these patients results from a complex interplay between the virus, the immune system, and the coagulation system. Studies have shown that the use of prophylactic anticoagulation therapy in COVID-19 patients significantly reduces the risk of venous thromboembolism, arterial thrombosis, and overall mortality [7]. However, like any therapy, prophylactic anticoagulation therapy is not without risks. The most significant risk associated with this therapy is an increased risk of bleeding, which must be carefully considered and monitored in each patient [8,9].

Overview of standard prophylactic anticoagulation therapy

Standard prophylactic anticoagulation therapy for COVID-19 patients typically involves using low-molecular-weight heparin (LMWH) or unfractionated heparin (UFH). LMWH is usually dosed based on the patient's weight, while UFH dose is monitored through activated partial thromboplastin time levels. This approach helps to ensure that patients receive appropriate anticoagulation therapy while minimizing the likelihood of adverse events [7].

Determining the duration of prophylactic anticoagulation therapy involves various factors, such as the patient's risk of thrombosis and bleeding and their clinical course. By adopting an individualized approach to determine the appropriate duration of therapy, healthcare providers can ensure that patients receive the necessary duration of anticoagulation therapy based on their specific circumstances. This tailored approach allows healthcare providers to optimize the benefits of anticoagulation therapy while minimizing the risk of adverse events. By administering LMWH or UFH and tailoring dosing and duration based on patient-specific factors, healthcare providers can help reduce the risk of thrombosis in patients with COVID-19 pneumonia. Additionally, an individualized approach can help minimize the risk of adverse events associated with anticoagulation therapy [7-9].

Benefits of HD-PA therapy

HD-PA therapy is a potential treatment option for COVID-19 pneumonia patients at an increased risk of developing thrombotic events. This therapy involves the administration of a higher dose of anticoagulant medication than the standard prophylactic therapy. For instance, 334 patients were randomly assigned to SD-PA and HD-PA, using LMWH for 14 days or until hospital discharge or weaning from supplemental oxygen for 48 consecutive hours outcome occurred first. The HD-PA patients received double the dose of SD-PA [4].

The mechanisms of action of HD-PA therapy are similar to those of standard prophylactic therapy. Anticoagulants work by inhibiting the coagulation cascade. Some act directly by binding and inhibiting the enzyme, while others act indirectly by binding to antithrombin or by preventing their synthesis from the liver (vitamin K-dependent factors). In this way, they prevent the formation of blood clots. However, high-dose prophylactic therapy is believed to be more effective in preventing thrombotic events than standard prophylactic therapy because it provides more anticoagulation [10].

Research has indicated that administering HD-PA therapy can lower the incidence of thrombotic events like venous thromboembolism (VTE) and others in patients with COVID-19 pneumonia. The evidence suggests that HD-PA therapy is a viable treatment option for COVID-19 pneumonia patients at a high risk of developing thrombotic events. A randomized controlled trial conducted by Tacquard et al. included 538 patients; 104 patients experienced 122 TCs with an incidence of 22.7%. Pulmonary embolism accounted for 52% of the recorded TCs. HD-PA was associated with a significantly reduced risk of TC without increasing the risk of hemorrhages or excessive bleeding [11].

Risks of HD-PA therapy

HD-PA therapy is associated with an increased risk of bleeding as a potential adverse effect. This risk is particularly relevant in patients with an underlying bleeding disorder or who have recently undergone major surgery. Bleeding can occur in various locations, including the gastrointestinal tract, urinary tract, and the brain, and may be life-threatening in some cases [4].

Another potential risk associated with HD-PA therapy is the development of heparin-induced thrombocytopenia (HIT). HIT is an immune-mediated disorder that can occur in response to exposure to heparin, which leads to a decrease in platelet counts and an increased risk of thrombosis [12-14]. HIT can lead to the formation of clots in small blood vessels, causing damage to organs, such as the kidneys, liver, and lungs, and can even lead to limb amputation [3]. The incidence of HIT with HD-PA therapy is relatively low, but it can have serious consequences for patients who develop this condition [12]. Therefore, while HD-PA therapy can effectively prevent thrombotic events in COVID-19 patients, clinicians must weigh the potential risks against the benefits of this therapy and carefully consider patient factors, such as bleeding risk and HIT history, before initiating treatment.

Patient selection for HD-PA therapy

The use of HD-PA therapy in COVID-19 pneumonia patients has demonstrated a promising effect in reducing the incidence of VTE and other thrombotic events. However, it is crucial to carefully identify the patients most likely to benefit from this therapy while also considering those at high risk of bleeding [11].

Selecting patients is a critical step in administering HD-PA therapy. Identifying patients at the highest risk of developing VTE and other thrombotic events is vital, as they are the most likely to benefit from the therapy. These patients may include those with pre-existing risk factors for thromboembolism, such as obesity, advanced age, and comorbidities like heart disease or cancer. Additionally, patients admitted to the ICU or requiring mechanical ventilation may also be at high risk for thrombotic events and would benefit from prophylactic anticoagulation therapy [14,15].

On the other hand, it is essential to consider patients at high risk of bleeding when considering HD-PA therapy. Such patients include those with a history of bleeding disorders or taking medications that increase the risk of bleeding. Patients who have undergone recent surgery or have experienced recent trauma are also at high risk for bleeding and may not be suitable candidates for this therapy [16]. Therefore, it is critical to carefully assess the patient's risk factors and weigh the potential benefits and risks before initiating HD-PA therapy.

Identification of Patients at High Risk of VTE and Thrombotic Events

Numerous risk factors have been identified as significant for developing VTE and other thrombotic events in COVID-19 pneumonia patients. Advanced age, obesity, immobility, prolonged hospitalization, and co-morbidities, such as diabetes, hypertension, and cardiovascular diseases, are associated with an increased risk of developing thrombotic events [17]. Advanced age has been identified as a major risk factor for VTE in COVID-19 patients, with older individuals being more likely to experience clotting events than their younger counterparts. Obesity has also been identified as a significant risk factor, with studies indicating that overweight individuals are at higher risk of developing thrombotic events than those of normal weight. Moreover, immobility, often a consequence of hospitalization, significantly contributes to developing thrombotic events in COVID-19 patients [18,19].

Other co-morbidities associated with an increased risk of VTE and thrombotic events in COVID-19 pneumonia patients include diabetes, hypertension, and cardiovascular diseases. These conditions are known to increase the risk of clotting events and can exacerbate the effects of COVID-19, leading to a greater likelihood of developing TCs [20]. Lastly, laboratory markers, such as elevated D-dimer and fibrinogen levels, predict thrombotic events in COVID-19 patients. Elevated levels of D-dimer, a fibrin degradation product indicative of ongoing thrombosis, have been found in a high proportion of COVID-19 patients with severe disease, suggesting a heightened risk of thrombotic events. Similarly, high fibrinogen levels, a protein that plays a key role in blood clotting, have been linked to an increased risk of TCs in COVID-19 patients [21,22].

Identification of Patients at High Risk of Bleeding

Patients at a high risk of bleeding may be at an increased risk of experiencing adverse events if they receive HD-PA therapy. Factors contributing to the likelihood of bleeding in COVID-19 pneumonia patients include having a history of bleeding disorders, renal impairment, liver dysfunction, and concurrently using other medications such as nonsteroidal anti-inflammatory drugs and antiplatelet agents that can increase bleeding risk [23].

Given this potential risk, it is crucial to carefully consider patient selection for HD-PA therapy and evaluate the balance between the risks and benefits of the treatment for each patient. In certain instances, alternative therapies or administering lower doses of anticoagulation may be more appropriate to minimize the potential for adverse events.

## Conclusions

HD-PA therapy has been shown to have potential benefits in reducing thrombotic events and lowering mortality rates in COVID-19 pneumonia patients. However, the therapy carries a higher risk of bleeding and HIT in some patients, necessitating carefully considering patient selection to balance the risks and benefits. Although the therapy shows promise, further research is needed to determine the optimal dosing, duration, and patient selection for HD-PA therapy in COVID-19 patients. Future studies should focus on identifying patients who are most likely to benefit from the therapy and assessing the long-term effects of the therapy and the risks of bleeding and HIT. Such research may refine HD-PA therapy in COVID-19 pneumonia patients and improve patient outcomes.
